# Oxidative Stress, Mitochondrial Dysfunction, and Aging

**DOI:** 10.1155/2012/646354

**Published:** 2011-10-02

**Authors:** Hang Cui, Yahui Kong, Hong Zhang

**Affiliations:** Department of Cell Biology, University of Massachusetts Medical School, Worcester, MA 01655, USA

## Abstract

Aging is an intricate phenomenon characterized by progressive decline in physiological functions and increase in mortality that is often accompanied by many pathological diseases. Although aging is almost universally conserved among all organisms, the underlying molecular mechanisms of aging remain largely elusive. Many theories of aging have been proposed, including the free-radical and mitochondrial theories of aging. Both theories speculate that cumulative damage to mitochondria and mitochondrial DNA (mtDNA) caused by reactive oxygen species (ROS) is one of the causes of aging. Oxidative damage affects replication and transcription of mtDNA and results in a decline in mitochondrial function which in turn leads to enhanced ROS production and further damage to mtDNA. In this paper, we will present the current understanding of the interplay between ROS and mitochondria and will discuss their potential impact on aging and age-related diseases.

## 1. Introduction

The fundamental manifestation of the aging process is a progressive decline in the functional maintenance of tissue homeostasis and an increasing propensity to degenerative diseases and death [1]. It has attracted significant interest to study the underlying mechanisms of aging, and many theories have been put forward to explain the phenomenon of aging. There is an emerging consensus that aging is a multifactorial process, which is genetically determined and influenced epigenetically by environment [2]. Most aging theories postulate a single physiological cause of aging, and likely these theories are correct to a certain degree and in certain aspects of aging. 

Reactive oxygen species (ROS) are highly reactive molecules that consist of a number of diverse chemical species including superoxide anion (O_2_^−^), hydroxyl radical (^·^OH), and hydrogen peroxide (H_2_O_2_). Because of their potential to cause oxidative deterioration of DNA, protein, and lipid, ROS have been implicated as one of the causative factors of aging [3]. As ROS are generated mainly as by-products of mitochondrial respiration, mitochondria are thought to be the primary target of oxidative damage and play an important role in aging. Emerging evidence has linked mitochondrial dysfunction to a variety of age-related diseases, including neurodegenerative diseases and cancer. Details of the precise relationship between ROS-induced damage, mitochondrial dysfunction, and aging remain to be elucidated.

## 2. ROS and Aging

### 2.1. ROS, Oxidative Damage, and Cellular Signaling

There are several sources of ROS within a cell. ROS are generated as by-products of aerobic respiration and various other catabolic and anabolic processes [4]. Mitochondria are the major producer of ROS in cells, and the bulk of mitochondrial ROS is generated at the electron transport chain [5, 6]. Electrons leak from the electron transport chain directly to oxygen, producing short-lived free radicals such as superoxide anion (O_2_^−^) [7, 8]. O_2_^−^ can be converted to nonradical derivatives such as hydrogen peroxide (H_2_O_2_) either spontaneously or catalyzed by superoxide dismutase (SOD) [9–13]. H_2_O_2_ is relatively stable and membrane permeable. It can be diffused within the cell and be removed by cytosolic antioxidant systems such as catalase, glutathione peroxidase, and thioredoxin peroxidase [14, 15]. In addition to being generated during cellular metabolism in mitochondria, ROS can be produced in response to different environmental stimuli such as growth factors, inflammatory cytokines, ionizing radiation, UV, chemical oxidants, chemotherapeutics, hyperoxia, toxins, and transition metals [16–26]. Other than mitochondrial respiration, a number of cytosolic enzymes are able to generate ROS [27]. The nicotinamide adenine dinucleotide phosphate (NADPH) oxidases are a group of plasma membrane-associated enzymes found in a variety of cell types [28]. The function of NADPH oxidases is to produce superoxide from oxygen using electrons from NADPH [29].

Once they are produced, ROS react with lipids, proteins, and nucleic acids causing oxidative damage to these macromolecules [30–34]. ROS readily attack DNA and generate a variety of DNA lesions, such as oxidized DNA bases, abasic sites, and DNA strand breaks, which ultimately lead to genomic instability [35]. 7,8-dihydro-8-oxo-deoxyguanosine (8-oxo-dG) is one of the most abundant and well-characterized DNA lesions caused by ROS [36]. It is a highly mutagenic lesion that results in G : C to T : A transversions [37]. To limit the cellular damage caused by ROS, mammalian cells have evolved a number of sophisticated defense mechanisms. ROS-generated DNA lesions are repaired mainly by base excision repair as well as other DNA repair pathways including nucleotide excision repair, double-strand break repair, and mismatch repair [38–40]. In addition, the damaging effects of ROS can be neutralized via elevated antioxidant defense, which includes superoxide dismutase, catalase, and glutathione peroxidase to scavenge ROS to nontoxic forms [41]. 

Intracellular ROS are normally maintained at low but measurable level within a narrow range, which is regulated by the balance between the rate of production and the rate of scavenging by various antioxidants [42]. ROS, at low level under normal conditions, is found to act as signaling molecules in many physiological processes, including redox homeostasis and cellular signal transduction [7]. By activating proteins such as tyrosine kinases, mitogen-activated protein kinases, or Ras proteins, ROS are important mediators of signal transduction pathways [7]. Dependent on cell types, ROS have been found to function as signaling molecules in cell proliferation [43], cellular senescence [44], or cell death [45, 46]. The divergent effects of ROS on many cellular processes suggest that ROS are not merely detrimental byproducts, but also generated purposefully to mediate a variety of signaling pathways.

### 2.2. The Free Radical Theory of Aging

The free radical theory of aging proposed by Denham Harman more than fifty years ago postulates that aging results from the accumulation of deleterious effects caused by free radicals, and the ability of an organism to cope with cellular damage induced by ROS plays an important role in determining organismal lifespan [3]. In agreement with this theory, increased ROS production by mitochondria and increased 8-oxo-dG content in the mtDNA are frequently detected in aged tissues [40, 47–50], suggesting that progressive accumulation of oxidative DNA damage is a contributory factor to the aging process. Consistently, many studies have found that increased oxidative damage in cells is associated with aging [51–53]. Furthermore, genetic studies in worm, fly, and mouse have linked enhanced stress resistance or reduced free radical production with increased lifespan [27]. Mutant strains of *C. elegans* that are resistant to oxidative stress have extended lifespan, whereas those more susceptible to free radicals have shortened lifespan [54, 55]. Mice lacking the antioxidant enzyme superoxide dismutase 1 (SOD1) exhibit a 30% decrease in life expectancy [56]. Conversely, simultaneous overexpression of SOD1 and catalase extends lifespan in Drosophila [57]. Small synthetic mimetics of SOD/catalase increase lifespan in *C. elegans* [58], while treatment of antioxidant drugs in mice increases the median lifespan up to 25% [59, 60]. Further supporting this hypothesis, mice lacking Ogg1 and Myh, two enzymes of the base excision repair pathway that repairs oxidative DNA damage, show a 50% reduction in life expectancy [61]. Collectively, these studies demonstrate that interplay between ROS and protective antioxidant responses is an important factor in determining aging and lifespan.

Despite a large body of evidence supporting the role of ROS in aging, the free radical theory of aging faces some challenges [62]. Mice heterozygous for superoxide dismutase 2 (*Sod*2^+/−^) have reduced manganese SOD (MnSOD) activity, increased oxidative damage, but normal lifespan [63]. Overexpression of antioxidant enzymes in mice, such as SOD1 or catalase, does not extend lifespan [64, 65]. The median lifespan of mice heterozygous of glutathione peroxidase 4 (Gpx4^+/−^), an antioxidant defense enzyme that plays an important role in detoxification of oxidative damage to membrane lipids, is significantly longer than that of wild-type mice, even though Gpx4^+/−^ mice show increased sensitivity to oxidative stress-induced apoptosis [66]. Studies of long-lived rodents also do not find a convincing correlation between level of oxidative damage and aging [67]. Furthermore, pharmacologic intervention with antioxidants in humans and mice has little effect on prolonging lifespan [68–70]. More investigations are clearly needed to clarify the discrepancy in the role of ROS and antioxidant enzymes in aging among different species and to understand the precise role that free radicals play in aging.

### 2.3. ROS and Senescence

Senescence, a process in which normal somatic cells enter an irreversible growth arrest after a finite number of cell divisions [71], is thought to contribute to organismal aging [72–74]. Senescent cells are associated with high level of intracellular ROS and accumulated oxidative damage to DNA and protein [75–77]. In contrast, immortal cells suffer less oxidative damage and are more resistant to the deleterious effects of H_2_O_2_ than primary cells [78]. Increasing intracellular oxidants by altering ambient oxygen concentrations or lowering antioxidant levels accelerates the onset of senescence, while lowering ambient oxygen or increasing ROS scavenging delays senescence [76, 78–81].

Telomere shortening is considered as the major cause of replicative senescence [82, 83]. It has been reported that the rate of telomere shortening is directly related to the cellular level of oxidative stress [84]. Telomere shortening is significantly increased under mild oxidative stress as compared to that observed under normal conditions, whereas overexpression of the extracellular SOD in human fibroblasts decreases the peroxide content and the rate of telomere shortening [79]. ROS can affect telomere maintenance at multiple levels. The presence of 8-oxoguanine (8-oxoG), an oxidative derivative of guanine, in telomeric repeat-containing DNA oligonucleotides has been shown to impair the formation of intramolecular G quadruplexes and reduces the affinity of telomeric DNA for telomerase, thereby interfering with telomerase-mediated extension of single-stranded telomeric DNA [85]. ROS also affect telomeres indirectly through their interaction with the catalytic subunit of telomerase, telomerase reverse transcriptase (TERT). Increased intracellular ROS lead to loss of TERT activity, whereas ROS scavengers such as *N*-acetylcysteine (NAC) block ROS-mediated reduction of TERT activity and delay the onset of cellular senescence [86]. Furthermore, the presence of 8-oxoG in the telomeric sequence reduces the binding affinity of TRF1 and TRF2 to telomeres [87]. TRF1 and TRF2 are components of the telomere-capping shelterin complex that protects the integrity of telomeres [88]. In addition, ROS-induced DNA damage elicits a DNA damage response, leading to the activation of p53 [89], a critical regulator of senescence. It has been shown that p53 transactivates E3 ubiquitin ligase Siah1, which in turn mediates ubiquitination and degradation of TRF2. Consequently, knockdown of Siah1 expression stabilizes TRF2 and delays the onset of replicative senescence [90]. The p53-Siah1-TRF2 regulatory axis places p53 both downstream and upstream of DNA damage signaling initiated by telomere dysfunction. By regulating telomere maintenance or integrity directly or indirectly, ROS plays a critical role in senescence.

### 2.4. ROS and Stem Cell Aging

Tissue-specific or adult stem cells, which are capable of self-renewal and differentiation, are essential for the normal homeostatic maintenance and regenerative repair of tissues throughout the lifetime of an organism. The self-renewal ability of stem cells is known to decline with advancing age [91–94], suggesting that decline in stem cell function plays a central role in aging. Increasing evidence suggests that dysregulated formation of ROS may drive stem and progenitor cells into premature senescence and therefore impede normal tissue homeostasis. 

Genetic studies of mice deficient in genes implicated in ROS regulation indicate that elevated level of ROS within the stem cell compartments leads to a rapid decline in stem cell self-renewal [95–98]. Deletion of Ataxia telangiectasia mutated (ATM) kinase results in increased ROS level in hematopoietic stem cell (HSC) population in aged mice, which correlates with a rapid decline in HSC number and function [95]. When *Atm* ^−/−^ mice are treated with antioxidants, the defect in stem cell self-renewal is rescued [95], suggesting that high level of ROS causes the decline in stem cell function. Furthermore, deficiency in telomerase reverse transcriptase (TERT) accelerates the progression of aging, resulting in an even shorter lifespan in *Atm* ^−/−^ mice accompanied by increased senescence in hematopoietic tissues and decreased stem cell activity [99]. These TERT-deficient HSCs are also sensitive to ROS-induced apoptosis, suggesting another possible cause of stem cell impairment during aging [99]. Similarly, defect in HSC number and activity accompanied by increased accumulation of ROS is observed in mice lacking three members of *Forkhead box O-class (FoxO)* [96–98]. Increased level of ROS in FoxO3-null myeloid progenitors leads to hyperproliferation through activation of the AKT/mTOR signaling pathway, and ultimately premature exhaustion of progenitors [100]. Mice carrying a mutation in *inner mitochondrial membrane peptidase 2-like* (*Immp2l*) gene, which is required to process signal peptide of mitochondrial cytochrome c1 and glycerol phosphate dehydrogenase 2, exhibit an early onset of aging phenotypes, including premature loss of fat [101]. Elevated mitochondrial ROS level in the *Immp2l* mutant mice leads to impaired self-renewal of adipose progenitor cells, suggesting that ROS-induced damage to adult stem cells is the driving force of accelerated aging in these mice [101]. Further supporting this notion, intracellular level of ROS is found to correlate with the long-term self-renewal ability of HSCs in mouse [102]. HSCs with high level of ROS show a decreased ability of long-term self-renewal, and treatment of antioxidant NAC is able to restore the functional activity of HSCs with high level of ROS [102]. Taken together, these studies suggest that ROS play an important role in stem cell aging. 

ROS-generated DNA lesions are repaired by several DNA repair pathways including base excision repair, nucleotide excision repair, double-strand break repair, and mismatch repair [38–40]. Endogenous DNA damage accumulates with age in HSCs in mouse. HSCs in mice deficient in DNA repair pathways, including nucleotide excision repair, telomere maintenance, and nonhomologous end-joining, exhibit increased sensitivity to the detrimental effect of ROS, diminished self-renewal and functional exhaustion with age [103]. These data support the notion that accumulated DNA damage is one of the principal mechanisms underlying age-dependent stem cell decline.

## 3. Mitochondria and Aging

### 3.1. The Mitochondrial Theory of Aging

Because mitochondria are the major producer of ROS in mammalian cells, the close proximity to ROS places mitochondrial DNA (mtDNA) prone to oxidative damage [104]. Consistently, many studies have shown that 8-oxo-dG, one of the common oxidative lesions, is detected at higher level in mtDNA than nuclear DNA, suggesting that mtDNA is more susceptible to oxidative damage [52, 105–113]. As both the major producer and primary target of ROS, mitochondria are thought to play an important role in aging. The mitochondrial theory of aging, extended from the free radical theory, proposes that oxidative damage generated during oxidative phosphorylation of mitochondrial macromolecules such as mtDNA, proteins, or lipids is responsible for aging [114]. As mtDNA encodes essential components of oxidative phosphorylation and protein synthesis machinery [115], oxidative damage-induced mtDNA mutations that impair either the assembly or the function of the respiratory chain will in turn trigger further accumulation of ROS, which results in a vicious cycle leading to energy depletion in the cell and ultimately cell death [104, 114, 116–118]. 

As mitochondria play a critical role in regulation of apoptosis, which is implicated in the aging process [119], age-related mitochondrial oxidative stress may contribute to apoptosis upon aging. The activation of the permeability transition pore in mitochondria, which is believed to play a critical role in cell necrosis and apoptosis, is enhanced in spleen, brain, and liver of aged mice [120, 121]. Moreover, mitochondrial adenine nucleotide translocase, a component of the permeability transition pore, exhibits an age-associated increase of oxidative modification in male houseflies [122]. Such selective oxidative modification may cause the cells more vulnerable to apoptotic inducers [123]. Thus, mitochondria appear to influence the aging process via modifying the regulatory machinery of apoptosis.

Mice expressing proof reading-deficient mitochondrial DNA polymerase show a consistent increase in mtDNA mutations, premature onset of the aging phenotypes and reduced lifespan [124, 125], suggesting a critical link between mitochondria and aging. Interestingly, ROS production in these mice is not increased [124, 125]. Similarly, mice expressing proof reading-deficient mitochondrial DNA polymerase specifically in heart show accumulation of mutations in mtDNA and develop cardiomyopathy, but oxidative stress in the transgenic heart is not increased, indicating that oxidative stress is not an obligate mediator of diseases provoked by mtDNA mutations [126]. More studies are required to further clarify the consequence of oxidative stress and mitochondrial dysfunction in aging.

### 3.2. Age-Associated Changes of Mitochondria

Mitochondrial genome encodes proteins required for oxidative phosphorylation and ATP synthesis, and RNAs needed for mitochondrial protein translation [115]. The mtDNA is densely packed with genes and only contains one noncoding region called the displacement loop (D-loop) [127]. The D-loop is important for mtDNA replication and transcription and has been extensively studied for the presence of age-related mutations [115]. Age-dependent accumulation of point mutations within the D-loop has been reported in various types of cells and tissues, including skin and muscle [128–132]. In addition to point mutations, deletions of mtDNA are detected at higher frequency in aged human and animal tissues [133–145]. Replication is thought to be the likely mechanism leading to the formation of mtDNA deletions [146–148], but recent studies suggest that mtDNA deletions may be generated during repair of damaged mtDNA rather than during replication [149]. It is thought that repair of oxidative damage to mtDNA accumulated during aging leads to generation of double-strand breaks [149], with single-strand regions free to anneal with microhomologous sequences on other single-stranded mtDNA or within the noncoding region [150]. Subsequent repair, ligation and degradation of the remaining exposed single strands would result in the formation of an intact mitochondrial genome harboring a deletion [149]. Whether and how exactly mutations and deletions of mtDNA cause the aging phenotypes are not clear. Among mtDNA deletions during aging, especially in postmitotic tissues like muscle and brain, the most common one is a 4977-bp deletion [151–153]. The frequency of this deletion increases in brain, heart, and skeletal muscle with age, although the increase varies in different tissues of the same individual [154], or even in different regions of the same tissue [134, 136, 137]. This deletion occurs in a region encoding subunits of the NADH dehydrogenase, cytochrome *c* oxidase, and ATP synthase [155]. Whether deletion of these genes plays a causative role in the development of aging phenotypes remains to be determined. 

In addition to age-associated increase of mtDNA mutations and deletions, the abundance of mtDNA also declines with age in various tissues of human and rodent [156–158]. For instance, in a large group of healthy men and women aged from 18 to 89 years, mtDNA and mRNA abundance is found to decline with advancing age in the vastus lateralis muscle. Furthermore, abundance of mtDNA correlates with the rate of mitochondrial ATP production [158], suggesting that age-related mitochondrial dysfunction in muscle is related to reduced mtDNA abundance. However, age-associated change in mtDNA abundance seems to be tissue specific, as several studies have reported no change in mtDNA abundance with age in other tissues in human and mouse [159–161]. It is possible that tissue-specific effect of aging on mtDNA abundance is related to the status of aerobic activity [156, 158], as aerobic exercise has been shown to enhance muscle mtDNA abundance in both human and mouse [162–164]. Increased prevalence of mtDNA mutations/deletions and decreased mtDNA abundance offer attractive underlying causes of mitochondrial dysfunction in aging, which warrants further investigation.

### 3.3. Mitochondria Malfunction in Age-Associated Human Diseases

A heterogeneous class of disorders with a broad spectrum of complex clinical phenotypes has been linked to mitochondrial defect and oxidative stress [165, 166]. Particularly, mitochondria are thought to play an important role in the pathogenesis of age-associated neurodegenerative diseases, such as Alzheimer's disease, Parkinson's disease, and Huntington's disease. This is not surprising as neurons are especially sensitive and vulnerable to any abnormality in mitochondrial function because of their high energy demand.

Alzheimer's disease (AD) is the most common form of dementia and often diagnosed in people over 65 years of age. AD is characterized by severe neurodegenerative changes, such as cerebral atrophy, loss of neurons and synapses, and selective depletion of neurotransmitter systems in cerebral cortex and certain subcortical region [167]. Mitochondria are significantly reduced in various types of cells obtained from patients with AD [168–170]. Dysfunction of mitochondrial electron transport chain has also been associated with the pathophysiology of AD [170]. The most consistent defect in mitochondrial electron transport enzymes in AD is a deficiency in cytochrome *c* oxidase [171, 172], which leads to an increase in ROS production, a reduction in energy stores, and disturbance in energy metabolism [173]. 

Parkinson's disease (PD) is the second most common progressive disorder of the central nervous system, which is characterized prominently by loss of dopaminergic neurons in the substantia nigra and formation of intraneuronal protein aggregates [174]. The finding that exposure to environmental toxins, which inhibit mitochondrial respiration and increase production of ROS, causes loss of dopaminergic neurons in human and animal models leads to a hypothesis that oxidative stress and mitochondrial dysfunction are involved in PD pathogenesis [175]. Consistent with this notion, a significant decrease in the activity of complex I of the electron transport chain is observed in the substantia nigra from PD patients [176]. Furthermore, neurotoxin 1-methyl-4-phenyl-1,2,3,6-tetrahydropyridine, which acts as an inhibitor of complex I, can induce parkinsonism in human, monkey, and rodent [177, 178]. Genetic studies of *PINK1* and *PARKIN* further support the role of mitochondrial dysfunction in pathogenesis of PD [179, 180]. Autosomal recessive mutations in *PINK1* and *PARKIN* are associated with juvenile Parkinsonism [181–183]. Studies in *Drosophila* have provided strong evidence that *PINK1* and *PARKIN* act in the same genetic pathway to control mitochondrial morphology in tissues with high energy demand and requirement of proper mitochondrial function, such as indirect flight muscle and dopaminergic neurons [184–186]. Consistent with the finding in *Drosophila*, primary fibroblasts derived from patients with *PINK1* mutations show similar abnormalities in mitochondrial morphology [187]. The morphologic changes of mitochondria can be rescued by expression of wild-type *PARKIN* but not pathogenic *PARKIN* mutants [187], suggesting that mitochondrial dynamics plays an important role in PD pathogenesis. 

Huntington's disease (HD) is another hereditary neurodegenerative disorder that affects muscle coordination and leads to cognitive decline and dementia. HD is caused by an autosomal dominant mutation in the Huntingtin (HTT) gene [188]. Morphologic defects of mitochondria, such as reduced mitochondrial movement and alterations in mitochondrial ultrastructures, have been observed in patients with HD or transgenic HD mouse models [189, 190]. In addition, expression of mutant HTT leads to impaired energy metabolism, abnormal Ca^2+^ signaling and mitochondrial membrane potential, and drastic changes in mitochondrial ultrastructures [191, 192]. Although the underlying molecular mechanism remains to be determined, it is recently proposed that mutant HTT conveys its neurotoxicity by evoking defects in mitochondrial dynamics, mitochondrial fission and fusion, and organelle trafficking, which in turn result in bioenergetic failure and HD-associated neuronal dysfunction [189].

Mitochondrial dysfunction and increased oxidative damage are often associated with AD, PD, and HD, suggesting that oxidative stress may play an important role in the pathophysiology of these diseases [193]. Increased production of cellular ROS and oxidative stress have been reported to induce autophagy, a homeostatic process that enables cells to degrade cytoplasmic proteins and organelles [194–197]. The observation of increased autophagy in the brains of patients with AD, PD, and HD suggests that autophagy contributes to the pathogenesis of these neurodegenerative diseases, possibly by causing cell death [170, 198–202]. Consistently, oxidative stress-induced autophagy of accumulated amyloid *β*-protein in AD causes permeabilization of lysosomal membrane and leads to neuronal cell death [203]. Mitochondria damaged by significantly increased oxidative stress in pyramidal neurons of AD are subjected to autophagic degradation, ultimately leading to neurodegeneration [204]. Furthermore, overexpression of wild-type PINK1 increases mitochondrial interconnectivity and suppresses toxin-induced autophagy, whereas knockdown of PINK1 expression potentiates mitochondrial fragmentation and induces autophagy [197], suggesting that induced autophagy as a consequence of loss of function of *PINK1* may contribute to the pathogenesis of PD. 

Interestingly, autophagy also serves as a protective mechanism in age-related neurodegenerative diseases. Several studies demonstrate that basal level of autophagy clears the deleterious protein aggregates that are associated with AD, PD, and HD [205–207], therefore playing a protective role in the maintenance of neural cells. For instance, autophagy is involved in degradation of HTT aggregates [198]. Administration of rapamycin induces autophagy and enhances the clearance of mutant HTT, improving cell viability and ameliorating HD phenotypes in cell and animal models [208]. Furthermore, PARKIN, whose loss of function mutation causes early onset PD, has been found to promote autophagy of depolarized mitochondria [209], suggesting that a failure to eliminate damaged mitochondria by mutant PARKIN is responsible for the pathogenesis of PD. It is not entirely clear why autophagy can exert protective or deleterious effects on pathogenesis of these neurodegenerative diseases. A better understanding of autophagy, mitochondrial dysfunction, and oxidative stress is necessary in order to dissect the pathogenesis of AD, PD, and HD. 

Cancer is considered an age-associated disease, as the incidence of cancer increases exponentially with age. Warburg first discovered that cancer cells constitutively metabolize glucose and produce excessive lactic acid even in the presence of abundant oxygen, a phenomenon named “aerobic glycolysis” [210]. In contrast, normal cells generate energy mainly from oxidative breakdown of pyruvate, which is an end product of glycolysis and is oxidized in mitochondria. Conversion of glucose to lactate only takes place in the absence of oxygen (termed “Pasteur effect”) in normal cells. He hypothesized that defect in mitochondrial respiration in tumor cells is the cause of cancer, and cancer should be interpreted as mitochondrial dysfunction [210]. A growing body of evidence has demonstrated the presence of both somatic and germline mutations in mtDNA in various types of human cancers [211–213]. The most direct evidence that mtDNA mutations may play an important role in neoplastic transformation comes from the study by introducing a known pathogenic mtDNA mutation T8993G into the prostate cancer cell line PC3 through transmitochondrial cybrids [214]. The T8993G mutation derived from a mitochondrial disease patient causes a 70% reduction in ATP synthase activity and a significant increase in mitochondrial ROS production [215]. Tumor growth in the T8993G mutant cybrids is much faster than that in the wild-type control cybrids [214]. Moreover, staining of tumor sections confirms a dramatic increase in ROS production in T8993G mutant tumors, suggesting that mitochondrial dysfunction and ROS elevation contribute to tumor progression. Consistent with this notion, the *Sod*2^+/−^ mice exhibit increased oxidative damage and enhanced susceptibility to cancer as compared to wild-type mice [63]. Collectively, these studies suggest that mtDNA mutations could contribute to cancer progression by increasing mitochondrial oxidative damage and changing cellular energy capacities.

### 3.4. Mouse Models of Oxidative Stress and Mitochondrial Dysfunction in Aging

Genetically engineered mouse models provide great systems to directly dissect the complex relationship between oxidative damage, mitochondrial dysfunction, and aging. Although it is difficult to manipulate mitochondrial genome, genetic engineering of nuclear genes that are involved in oxidative stress response and mitochondrial function has been utilized to study mitochondrial biology and aging. 

Mammalian cells scavenge ROS to nontoxic forms through a sophisticated antioxidant defense that includes superoxide dismutase (SOD), catalase, and glutathione peroxidase. Genetic ablation of *SOD*2, which encodes a mitochondrial manganese SOD (MnSOD), leads to early postnatal death in mice accompanied by a dilated cardiomyopathy, metabolic acidosis, accumulation of lipid in liver and skeletal muscle, increased oxidative damage, and enzymatic abnormalities in mitochondria [216, 217]. Treatment of *Sod*2^−/−^ mice with a synthetic SOD mimetic not only rescues their mitochondrial defects in the liver, but also dramatically prolongs their survival [218]. Furthermore, heterozygous *Sod*2^+/−^ mice show evidence of decreased membrane potential, inhibition of respiration, and rapid accumulation of mitochondrial oxidative damage [219]. Mitochondrial oxidative stress induced by partial loss of *SOD*2 leads to an increase in proton leak, sensitization of the mitochondrial permeability transition pore and premature induction of apoptosis [219]. These studies clearly demonstrate that ROS generated in mitochondria play an important role in cell homeostasis and aging.

Conflicting results of the effect of increased *SOD2* expression on aging are obtained using different *SOD*2 transgenic mouse strains [220–222]. A transgenic line carrying a human SOD2 transgene under the control of a human *β*-actin promoter shows protection against hyperoxic lung injury [220], reduction in mitochondrial superoxide in hippocampal neurons, and extended lifespan as the result of increased activity of MnSOD [221]. Another transgenic line carrying a 13-kb mouse genomic fragment containing *SOD*2 [223] has a twofold increase in the activity of MnSOD [222]. Such level of *SOD*2 overexpression does not alter either lifespan or age-related pathology, even though these mice exhibit decreased lipid peroxidation, increased resistance against paraquat-induced oxidative stress, and decreased age-related decline in mitochondrial ATP production [222]. The reason behind the different outcomes of these two *SOD*2 transgenic mice on lifespan is not clear, but may be related to different levels of *SOD*2 expression. The precise role of SOD2 in aging needs further investigation. 

An important function of mitochondria is to produce ATP. Targeting genes involved in ATP production offers a great opportunity to study the role of mitochondrial function in aging. An example is a mouse model with targeted inactivation of adenine nucleotide translocator (ANT), a transporter protein that imports ADP and exports ATP from the mitochondria. *Ant*1^−/−^ mice exhibit classical physiological features of mitochondrial myopathy and hypertrophic cardiomyopathy in human, as evident of cardiac hypertrophy, an increase in succinate dehydrogenase and cytochrome *c* oxidase activities, a degeneration of the contractile muscle fibers, and a massive proliferation of abnormal mitochondria in skeletal muscle [224]. The increase in mitochondrial abundance and volume in muscle of *Ant*1^−/−^ mice is accompanied by upregulation of genes that are known to be involved in oxidative phosphorylation [225]. Consistently, mitochondrial H_2_O_2_ production increases in skeletal muscle and heart of *Ant*1^−/−^ mice [226]. The *Ant*1-deficient mouse model provides strong evidence that a defect in mitochondrial energy metabolism can result in pathological disease [224]. 

IMMP2L protein is a subunit of a heterodimer complex of inner mitochondrial membrane peptidase that cleaves signal peptide from precursor or intermediate polypeptides after they reach the inner membrane of mitochondria [227, 228]. Mammalian IMMP2L has two known substrates, cytochrome c1 and glycerol phosphate dehydrogenase 2, both of which are involved in superoxide generation [229]. The *Immp2l* mutant mice have impaired processing of signal peptide of cytochrome c1 and glycerol phosphate dehydrogenase 2 [230], and consequently show elevated level of superoxide ion, hyperpolarization of mitochondria, and increased oxidative stress in multiple organs. Furthermore, these *Immp2l* mutant mice exhibit multiple aging-related phenotypes, including wasting, sarcopenia, loss of subcutaneous fat, kyphosis, and ataxia [101]. These data provide a strong evidence that mitochondrial dysfunction is a driving force of accelerated aging.

## 4. Conclusion

Aging is a complex process involving a multitude of factors. Many studies have demonstrated that oxidative stress and mitochondrial dysfunction are two important factors contributing to the aging process. The importance of mitochondrial dynamics in aging is illustrated by its association with a growing number of age-associated pathogenesis. A better understanding of response to oxidative stress and mitochondrial dynamics will lead to new therapeutic approaches for the prevention or amelioration of age-associated degenerative diseases.
